# Two Birds with One Stone: Possible Dual-Role of Oxytocin in the Treatment of Diabetes and Osteoporosis

**DOI:** 10.3389/fendo.2015.00121

**Published:** 2015-08-10

**Authors:** Seham Elabd, Ismail Sabry

**Affiliations:** ^1^Human Physiology Department, Medical Research Institute, Alexandria University, Alexandria, Egypt; ^2^Zoology Department, Faculty of Science, Alexandria University, Alexandria, Egypt

**Keywords:** oxytocin, diabetes, insulin sensitivity, glucose uptake, osteoporosis, bone formation, bone resorption

## Abstract

Oxytocin (OT), a hormone most commonly associated with labor and lactation, may have a wide variety of physiological and pathological functions, which makes OT and its receptor potential targets for drug therapy. In this review, we highlight the newly discovered metabolic role of OT in diabetes and its complication, such as diabetic osteopathy. OT may have positive metabolic effects; this is based on the change in glucose metabolism, lipid profile, and insulin sensitivity. It may modify glucose uptake and insulin sensitivity both through direct and indirect effects. It may also cause regenerative changes in diabetic pancreatic islet cells. Moreover, it has an anabolic effect on the bone biology. So, the activation of the OT receptor pathway by infusion of OT, OT analogs, or OT agonists may represent a promising approach for the treatment of diabetes and some of its complications, including diabetic osteopathy.

## Introduction

The story of oxytocin (OT) begins right before labor, continues during birth, and later during the early period of lactation. Several physiological and pathological functions are governed directly or indirectly by OT (1, 2). This makes OT, and its receptor, potential targets for drug therapy. The therapeutic use of the OT is limited to, initiate labor and milk ejection (1, 2). However, there are indications that show a significant role of OT away from pregnancy (1, 2). OT is considered as a cardiovascular hormone with cardio-protective effects under experimental conditions (3). It has been shown that cardiovascular dysfunctions are related to metabolic disorders, such as obesity, insulin resistance, and diabetes (4).

Diabetes is one of the metabolic disorders considered as one of the major causes of mortality worldwide. Its incidence is expected to increase dramatically over the coming decades (5). It is characterized by a chain of complications that affect many organs. Osteoporosis (diabetic osteopathy) is one of these complications (6). Many endogenous and exogenous factors, more or less dependent on each other, are known to influence energy metabolism and bone mass accumulation. OT is one of these endogenous factors. Several lines of evidence have largely demonstrated that OT has many positive metabolic effects; this is based on a remarkable change in glucose metabolism, lipid profile, and insulin sensitivity after OT administration (7–10). In addition, it has also an anabolic effect on bone metabolism (11–13).

## Metabolic Effect of OT

Feeding behavior, body weight, and energy balance are crucially regulated by the hypothalamus in the CNS (14). Several studies have linked OT to the hypothalamus–brainstem circuits that negatively regulate feeding (15–17). However, the physiological relevance and pathological relevance of OT were unexplored until researchers found that obesity linked to OT release defect, and OT treatment was able to effectively correct overeating and obesity (8–10). Deficits in OT or its receptor developed hyperleptinemia and late-onset obesity with increases in abdominal fats and fasting plasma triglycerides (TGs) (18, 19).

Oxytocin has a role in affecting glucose and insulin homeostasis, as well as regulating body weight balance. It has been reported that OT promotes glucose uptake (20, 21) and stimulates insulin secretion (22–26). It stimulated insulin secretion in pancreatic islets independently of plasma glucose changes while it also stimulated pancreatic glucagon secretion (25). This might suggest the involvement of OT in pathophysiology of diabetes. Recent research demonstrated that OT administration reduced obesity-related diabetic changes, such as glucose intolerance, insulin resistance, and pancreatic islet hypertrophy (7–10, 27, 28). Two weeks treatment with OT decreased adiposity and food intake in obese mice lacking leptin, although it worsen glucose metabolism, most likely due to an increase in corticosterone levels and enhanced hepatic glucose production. It could be suggested that the effect of OT in decreasing fat mass is independent of leptin, while the beneficial impact on glucose metabolism requires the presence of leptin (29). Whereas, OT treatment for longer period, notably reduced body fat accumulation, fasting blood glucose levels, and improved insulin sensitivity and glucose tolerance in leptin receptor deficient mice (30). The hypoglycemic effect, stimulatory effect on insulin secretion and sensitivity, and improvement of pancreatic islet cells after OT administration, strongly suggested that OT might be a therapeutic target for treating diabetes.

## Role of Oxytocin Signaling on Glucose and Lipid Metabolism

### Coupling of oxytocin receptors to intracellular cascades

Oxytocin receptor (OTR) is known to be present in neurons. This receptor is also present in other tissues like adipose tissue and pancreas (31). OT receptor is a G protein-coupled receptor (GPCR), which linked through G_αq/11_ to phospholipase C (PLC) (32). Activation of PLC causes an increase in inositol trisphosphate (IP3) and diacyl glycerol (DAG). IP3 activates specific receptors in the sarcoplasmic reticulum in order to release Ca^2+^ into the cytosol to form a complex with calmodulin. This activates myosin light chain (MCL) kinase to phosphorylate MLCs causing myocyte contraction (33). DAG induces protein kinase C (PKC) activation, which has been implicated in uterine contraction. OT receptor activation leads to an increase in prostaglandin production through phospholipase A2 and cyclooxygenase-2. The MAP-kinase (MAPK) cascade is activated by different pathways like *trans*-activation of receptor tyrosine kinases and also by possibly different G protein-linked pathways (34). In fact, the coupling of the OTR to Gs and Gi proteins is also occurring. The trophic effect of OT is occurring via a PKC-mediated activation of eukaryotic elongation factor-2 (35). In different cell systems, the multiple signaling pathways are activated by OTRs, which may act synergistically like contraction of myometrial cells that induced by OTR coupling to G_αq-11_ and to the small G proteins of the Rho pathway (36). However, they may also have an opposite effect on the same cell function, such as the proliferative effects of OT, which appear to be G_q_-linked and involve MAPK activation which leads to c-fos and c-jun induction. On the other hand, inhibition of cell growth has been reported to be G_i_ mediated (37). Due to this heterogeneity in the final outcome, functional selective ligands would be of great importance in identifying the roles of the different OTR-elicited pathways in physiological functions.

Arginine-vasopressin (AVP) is another posterior pituitary hormone, which differ from OT only by two amino acids. AVP and OT trigger their effects through at least four subtypes of receptors (V_1a_, V_1b_, V_2_, and OT receptors). V_1a_ receptors mediate glycogenolysis and vasoconstriction, V_lb_ receptors mediate the release of adrenocorticotropic hormone (ACTH), catecholamine, insulin, and glucagon, and V_2_ receptors mediate antidiuresis (38). Because OT and AVP share similar structures, it is not unexpected that they cross-react with each other’s receptor. For instance, AVP stimulates myometrial contraction via OTR (39) and OT stimulates the release of ACTH through V_1b_ (40). OT interacts with AVP receptors with a lower affinity. It has been reported that both OT and AVP induce insulin and glucagon release from the pancreas by activating V_1b_ receptors (41, 42).

### Glucose and insulin homeostasis

**Figure 1:**
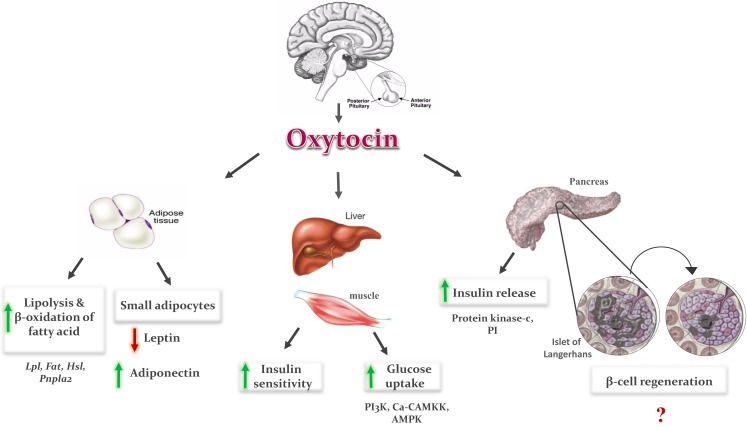
**Metabolic effects of oxytocin: OT is secreted from the posterior lobe of the pituitary gland and binds to its receptor in peripheral tissues**. In adipose tissue, it induces fatty acid oxidation and lipolysis, and formation of small adipocytes. Small adipocytes increase secretion of adiponectin and decrease leptin secretion, which improve insulin sensitivity in adipose tissue, liver, and muscles. In pancreas, it induces insulin secretion via phosphoinositide (PI) turnover and activation of protein kinase C, and regeneration of pancreatic β-cells. In liver and muscles, it enhances glucose uptake by stimulation of intracellular release of calcium, and activation of phosphoinositid-3-kinase (PI3K), calcium-calmodulin kinase kinase (Ca-CAMKK), and AMP-activated protein kinase (AMPK).

Oxytocin can be a good choice to decrease the blood glucose level and increase the insulin level. The hypoglycemic effect of OT can be explained by increasing glucose uptake via insulin-like signaling pathway (20, 21). The glucose uptake in cardiomyocytes (CMs) is increased by OT through stimulation of intracellular release of Ca^2+^, and also activation of phosphoinositide-3-kinase (PI3K), calcium-calmodulin kinase kinase (Ca-CAMKK), and AMP-activated protein kinase (AMPK) pathways (Figure 1) (20, 21). The hypoglycemic actions of OT are also believed to be mediated indirectly through changes in pancreatic functions (25) as OT modulates insulin secretion centrally via vagal cholinergic neurons innervating β-cells and peripherally via phosphoinositide turnover and activation of protein kinase-C in pancreatic β-cells (Figure 1) (22–26).

Pancreatic islet inflammation is an important factor in the pathogenesis of diabetes. The protection of β-cells from death is considered as a new therapeutic target. This inflammatory process is probably a combined consequence of dyslipidemia, hyperglycemia, and increased circulating proinflammatory cytokines [leptin, interleukin-1 (IL-1), interleukin-6 (IL-6), and tumor necrosis factor-α (TNF-α) (43)]. OT decreases pancreatic islet hypertrophy (7, 28). Yet, the remaining question is whether OT directly promotes β-cell regeneration or it acts indirectly. Unfortunately, the exact mechanism is still unknown and further studies are needed. Whereas, the indirect effect(s) may be related to its action as insulin sensitizers, with a reduction in gluco- and lipotoxicity (7–10). As well as, OT has antioxidant and anti-inflammatory effects (44–46).

In addition to increasing insulin secretion, OT also improves insulin sensitivity by both reducing gluco- and lipotoxicity (7–10) and regulating cytokines like leptin and adiponectin (7, 9). OT decreased fat mass, resulting in reduction in leptin level, which could also be one of the reasons underlying the OT-induced improvement in insulin sensitivity (9). However, the results of adiponectin after OT administration are contradictory. In some studies, adiponectin concentration was not affected by OT administration (30, 47), and in others, OT treatment induces an increase in adiponectin level (7). OT treatment induced formation of small adipocytes in treated rats as compared to control (47). It could be the smaller adipocytes, which are more sensitive to insulin produce a lower amounts of leptin and higher amounts of adiponectin (Figure 1). The balance of leptin and adiponectin in diabetic patients can be used as a predictor of insulin resistance and a useful indicator for the choice of drug to treat diabetes mellitus (48).

### Lipid metabolism

Oxytocin improves lipid profile of diabetic rodents as well by lowering serum low-density lipoprotein (LDL), TG, and cholesterol levels, and a propensity for high-density lipoprotein (HDL) level improvements (7). OT induces lipolysis and β-oxidation of fatty acids (9, 10). The effects of OT on adipocytes are mediated either directly by a mechanism via its peripheral effects on adipocytes, or indirectly through the central anorectic action and centrally mediated activation of sympathetic nerve innervating fat tissue. Peripheral actions of the OT are mediated by OTR. OT receptor mRNA is highly expressed in mouse adipocytes and increased during the differentiation of adipocyte (49). The stimulatory effect of OT on lipid metabolism is mediated by an increase in the mRNA expression of lipoprotein lipase (*Lpl*) and fatty acid transporter (*Fat*, also known as CD36). These two enzymes are responsible for the uptake of circulating TG and fatty acids, respectively. On the other hand, OT does not modify the mRNA level of enzymes involved in lipogenesis and TG storage, such as acetyl-coenzyme A carboxylase α (*Acaca*, also known as ACC-α), fatty acid synthase (*Fasn*), and diacylglycerol *O*-acyltransferase homolog 1 (*Dgat1*). OT also increases the mRNA levels of the enzymes involved in the process of lipolysis, such as hormone-sensitive lipase (*Hsl*) and patatin-like phospholipase domain containing 2 (*Pnpla2*). The increase in lipolysis process and TG uptake without a change in lipogenesis and TG storage under the effect of OT shows that OT promotes the use of fat as energy substrate (9). OT treatment induces formation of small adipocytes in treated rats as compared to control (47). This adipogenic effect of OT could be explained by the activation of peroxisome proliferator-activated receptors-γ (PPARγ), a key regulator of adipocyte differentiation (50).

## Anabolic Actions of OT on the Skeleton

One of the most important bone-research results has revealed that the pituitary hormones have an intense outcome on bone (51), so that the pituitary–bone axis turned into one of the most important topics in skeletal physiology. Based on the fact that calcium is mobilized from the maternal skeleton during lactation and late pregnancy, it might be expected that the same hormone that regulates parturition and lactation might also controls skeletal homeostasis. Our research group and other scholars have developed studies to find new bone formation therapies. As a primitive neurohypophyseal hormone, OT has been reported to be an anabolic bone mass regulator (11–13).

A specific and accurate method for measuring OT is urgently needed for a better understanding of OT role, because the methods that are currently used to measure the peripheral OT level lack reliability (52). Previous study reported that plasma levels of OT are notably lower in postmenopausal women who develop osteoporosis than in healthy peers (12). Changes in bone remodeling and osteopenia in ovariectomized (OVX) rodents resulted in a significantly decreased OT level when it is compared with sham-operated controls (53). Moreover, in genetically modified animals, it was confirmed that deletion of OT or OTR causes a remarkable reduction in bone formation and this suggested that OT is crucial for basal skeletal homeostasis (13). Interestingly, it has been found that OT treatment in mice increased bone mineral density as well as osteoblast formation (11), it also reversed bone loss in 8-week-old OVX mice, and reduced marrow adiposity (12, 13). Its positive bone balance suggests that OT could be used therapeutically as an ally in the recovery of osteopathy resulting from diabetes.

## Role of Oxytocin Signaling on Bone Metabolism

**Figure 2:**
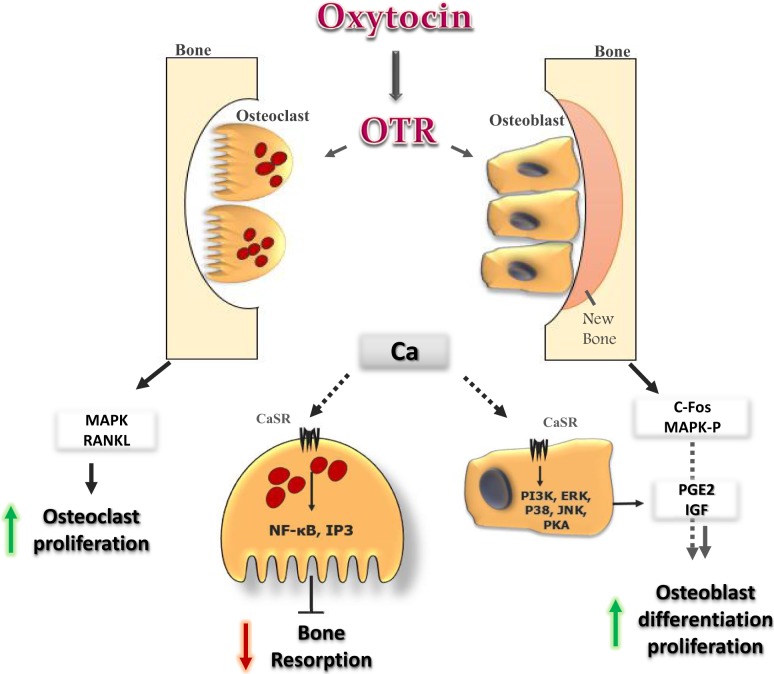
**Oxytocin signaling in bone cells: OT binds to its receptor in osteoclast (OC) and osteoblast (OB), and initiates several cellular cascades**. It induces OB differentiation by inducing c-fos expression and MAP-kinase phosphorylation. OT induces release of Ca^2+^ from intracellular stores. In OB, the increase in Ca^2+^ provokes several cellular cascades (JNK, P38, ERK, PKA, and PI3K), which lead to an increase in prostaglandin E2 synthesis, with positive effects on OB. In OC, OT increases OC formation directly, by activating MAPK signaling, and indirectly through the up-regulation of RANK-L from OB. The increase in Ca^2+^ induces NF-kb and IP3 which inhibit bone resorption of mature OC.

At the cellular level, OT stimulates differentiation of osteoblasts to a mineralizing phenotype by up-regulating bone morphogenic protein-2 (13). The effect of OT on osteoclasts was found to be more complex as osteoclast formation was stimulated, while bone resorption by mature osteoclasts was inhibited (7, 54). The effect of OT on bone could be resulted from the direct interaction of the hormone with its receptors in bone cells (54, 55). The signaling cascades that mediate the OT action on bone cells eventually release of Ca^2+^ from intracellular stores and trigger MAPK signaling (13). The increase in intracellular Ca^2+^ in osteoblasts provokes cellular cascades, such as JNK, P38, extracellular signal regulated kinase (ERK), PKA, and PI3K (56). This leads to an increase in prostaglandin E2 synthesis, with subsequent positive bone balance. OT and OTR interactions in osteoblast may also provoke other intracellular events, such as MAP-kinase phosphorylation and induce c-Fos expression (Figure 2) (55). It has been reported that OTR, on stimulation by its ligand OT, translocates into the nucleus of osteoblasts (57). The passage of OTR into the nucleus is facilitated by successive interactions with β-arrestins (ARRBS), the small GTPase Rab5, importin-β (KPNB1), and transportin-1 (TNPO1). This mechanism for OTR translocation will open several possibilities for direct transcriptional modulation by nuclear GPCRs (57). While in osteoclast, OT has dual effects. It increases osteoclast formation both directly, by activating nuclear factor-kβ and MAPK signaling, and indirectly via the up-regulation of receptor activator of nuclear factor kappa-B ligand (RANK-L) (Figure 2). The increase in osteoclastogenesis is coupled to the decrease in bone resorption of mature osteoclasts and this can be explained by triggering the cytosolic Ca^2+^ release and nitric oxide synthesis (13, 54). This might explain the significant decrease in the levels of TRAP, a bone-resorption marker that observed after OT administration (7). In addition to OTR, both AVP receptors, V_1a_ and V_2_, are expressed in osteoblasts and osteoclasts, and their stimulation induces ERK activation, which in turn inhibits bone formation and stimulates bone resorption. This coupling would lead to bone loss (58). So, it could be concluded that the anabolic effects of OT on bone are not occurring through activation of vasopressin receptor signaling.

In addition to its direct effect, OT was found to have an indirect effect on bone through its action on cytokines (leptin and adiponectin) (7, 9) that have a considerable role in bone biology. Leptin has two opposite effects on bone. Centrally, it suppresses bone formation by restraining osteoblast proliferation. Peripherally, it stimulates osteoblastic differentiation and inhibits osteoclastogenesis. The two pathways could compensate each other, with the peripheral and positive effects being the major when leptin central resistance occurs with obesity onset (59). Adiponectin stimulates the differentiation and mineralization of osteoblasts and inhibits the macrophage colony-stimulating factor (M-CSF)- and RANKL-induced osteoclast differentiation, as well as the bone-resorption activity of mature osteoclasts (60).

## Exogenous Oxytocin Administration

Besides its use in obstetrics, it has been reported that OT could play an important role in treating psychiatric disorders, such as autism and schizophrenia (61, 62). The most precise and reliable mode of delivering OT is through infusing it directly into the blood. Orally administered OT, due to its peptide structure, is rapidly degraded by proteolytic enzymes in the gut. Plasma-delivered OT, such as an intravenous injection, does not cross the blood–brain barrier. Intranasal OT is currently the favored administration method for neurobehavioral research because of its absorption through the nasal mucosa. Efficient brain penetration and activation of OTR are potentially an important limitation to the current OT therapies. Yet, the next generation of therapeutics may bypass this problem. Pharmacologically enhancing endogenous OT release, or developing small-molecule agonists and positive allosteric modulators, may be more effective (2). Regarding therapeutic consequences, the coupling of OTR to different G proteins exhibiting opposite effects, renders the definition of “agonist” and “antagonist” rather questionable. The ability to design compounds that can discriminate between many pathways, and predominantly activate a specific intracellular reaction, will be at the heart of future OTR-based drug strategies (2). Taking into consideration a potential use of OT as a therapeutic agent, an important issue to be addressed is the possible side effects of this treatment. OT’s side effect profile appears relatively benign according to recent studies focused on its effects on children and adolescents. No severe side effects of any kind, metabolic, or otherwise were reported (61, 62), but larger studies are still needed.

## Conclusion

These beneficial observations, which are discussed above, clearly showed that OT and its analogs are involved in regulation of metabolic and bone balance and are opening up a new window for drug development in diabetes and osteoporosis therapeutics. The potential therapeutic uses of OT and more long-acting and specific analogs of OT are huge. As a short nine amino acid peptide, OT is considered to be ideal for the design of agonists and antagonists of its receptor. Continued studies are still needed to develop new drugs including the use of OT, OT agonists, and antagonists for various human disorders including diabetes and osteoporosis.

## Conflict of Interest Statement

The authors declare that the research was conducted in the absence of any commercial or financial relationships that could be construed as a potential conflict of interest.
